# Diagnostic value of the cardiophrenic lymph nodes in gastric peritoneum metastases

**DOI:** 10.4314/ahs.v25i1.25

**Published:** 2025-03

**Authors:** Xiaolong Gu, Yang Li, Gaofeng Shi, Li Yang, Yang Yang, Zhidong Zhang

**Affiliations:** 1 Department of Radiology, The Fourth Hospital of Hebei Medical University, Shijiazhuang 050000, China; 2 Department of Reproductive Medicine, The Second Hospital of Hebei Medical University, Shijiazhuang 050000, China; 3 The Third Department of Surgery, The Fourth Hospital of Hebei Medical University, Shijiazhuang 050000, China

**Keywords:** Cardiophrenic lymph nodes, Gastric cancer, Peritoneum metastases, Exact diagnosis

## Abstract

**Background:**

The presence of cardiophrenic lymph nodes (CPLN) was potentially associated with peritoneal involvement in patients with abdominal malignancies. In this study, gastric cancer patients were investigated for the relationship between CPLN and peritoneum metastases.

**Methodology:**

Peritoneal exploration was performed on 516 gastric cancer patients between 2017 and 2019, including 134 (26%) with peritoneal metastases (PM). An univariate and multivariate analysis of preoperative computed tomography (CT) scans was conducted to assess the association between CPLN and confirmed PM.

**Results:**

Among the factors associated with CPLN in the univariate analysis, PM accounted for the strongest association (P < 0.001). In the multivariate analysis, PM was the only independent factor associated with CPLN (odds ratio [OR], 7.8; 95% confidence interval [CI], 4.6 ∼ 13.0; P < 0.001). CPLN was significantly more common in the 38 patients with classic signs of PM compared to the 96 patients without visible signs of PM (36/38 [95%] versus 76/96 [79%]). CT scan sensitivity rose from 28% (38/134 patients) to 85% (114/134 patients) with CPLN detection added to other diagnostic signs of PM.

**Conclusion:**

Detecting CPLN on CT was a valuable tool for diagnosing peritoneal metastases of gastric cancer.

## Introduction

In terms of cancer deaths, gastric cancer (GC) ranks fourth among all types of cancer^1^. Peritoneal metastases (PM) affect a large number of gastric cancer patients^2^. Computed tomography (CT) has been the preferred method for diagnosing gastric cancer, but it is not sensitive enough to detect peritoneal metastasis. Even when CT scans specifically target isolated small peritoneal metastatic nodules during abdominal exploration, meaningful direct CT features may not be evident, especially in cases where typical metastatic signs are absent. A new radiomics model has shown promising potential in addressing this issue^3^-^5^. However, this model requires image delineation and input into a computer for analysis, making it less intuitive for radiologists to interpret by simply reading the films. Additionally, different research institutions have yielded varying results regarding whether the radiomics model is superior to the clinical model^6^. Other imaging methods, such as PET/CT with new imaging agents, have shown some value in improving the sensitivity of peritoneal metastasis diagnosis^7^. However, their high cost makes widespread application challenging.

Diagnostic laparoscopy has been used for earlier detection of simultaneous peritoneal metastases, but it remains an invasive procedure^8^. The presence or absence of peritoneal metastasis affects the treatment strategies for gastric cancer patients. Intraperitoneal chemotherapy has become an essential component of combined treatment for gastric cancer, and ongoing translational research aims to address this specific disease state^9^.

Previous studies have suggested that the presence of cardiophrenic lymph nodes (CPLN) may be potentially associated with peritoneal involvement in patients with abdominal and pelvic malignancies^10^-^12^. CPLN could serve as a meaningful predictive marker in conditions such as ovarian cancer and colorectal cancer due to their distinct imaging features, allowing for direct interpretation by radiologists. Therefore, further investigation into the relationship between CPLN and peritoneal metastasis in patients with gastric cancer is warranted. This study analyzed preoperative CT scans of gastric cancer patients who underwent abdominal exploratory surgery to examine the relationship between CPLN and peritoneal metastasis.

In terms of cancer deaths, gastric cancer (GC) ranked fourth^1^. Peritoneal metastases (PM) affectd large numbers of gastric cancer patients^2^. Computed tomography (CT) was the preferred method for gastric cancer patients, but CT was not sensitive to of gastric cancer peritoneal metastasis. For some gastric cancer peritoneal metastases lacking typical metastatic signs, even if the CT images of isolated small peritoneal metastatic nodules were targeted after abdominal exploration, meaningful direct CT features could not be found. The new radiomics model demonstrated a promising approach^3^-^5^. However, it needed to delineate the image and inputed it into the computer to realize it, and it could not be judged intuitively by radiologists by reading the film, and different research institutions had different research results on whether the radiomics model was better than the clinical model^6^. Other imaging methods, such as PET/CT focusing on new imaging agents, had some value in terms of improving the sensitivity of diagnosing peritoneal metastases^7^, but their high cost maked them difficult to apply universally. Diagnostic laparoscopy for earlier diagnosis of simultaneous peritoneal metastases^8^, but it was still an invasive procedure. However, for gastric cancer patients, whether there was peritoneal metastasis or not, the treatment strategies were different. In recent years, intraperitoneal chemotherapy had become an indispensable part of the combined treatment of gastric cancer^2^,^9^, and some translational research was also aimed at addressing this specific disease state^8^.

Previous studies had shown that the presence of cardiophrenic lymph nodes (CPLN) was potentially associated with peritoneal involvement in patients with abdominal and pelvic malignancies, which might serve as a meaningful predictive marker, such as ovarian cancer^10^-^12^ and colorectal cancer^13^. And because the imaging features of CPLN were clear, they could be directly interpreted by radiologists. Therefore, the relationship between the preence of CPLN and peritoneal metastasis in patients with gastric cancer deserved further study.

Preoperative CT scans of gastric cancer patients undergoing abdominal exploratory surgery were analyzed. In this study, gastric cancer patients were investigated for the relationship between CPLN and peritoneum metastases.

## Methods

### Patient Population and Study Design

This retrospective study was approved by the ethics committee (protocol number 2021KY120); written informed consent was waived owing to the use of deidentified retrospective data. Our study retrospectively analyzed all consecutive GC patients who underwent surgery in our center between 2017 and 2019. The inclusion criteria were: (1) histologically confirmed gastric adenocarcinoma patients, (2) CT scan within two weeks before surgery, and (3) the peritoneal cavity had been completely explored during surgery. The exclusion criteria were: (1) prior anticancer therapy, (2) insufficient clinical data or CT images, and (3) patients with another cancer.

### Patient characteristics

Demographic characteristics such as age and gender were collected for the patients. Clinical features related to the tumor were obtained based on the surgeon's final evaluation. These features included the location of gastric adenocarcinoma, T stage (reflecting the depth of tumor invasion into the gastric wall), N stage (indicating the presence of gastric lymph node metastasis), and M stage (which encompassed peritoneal metastasis and other distant metastases).

Peritoneal metastasis was determined based on a comprehensive surgical report of peritoneal cavity exploration. Additionally, cytological examination of peritoneal lavage fluid was conducted for all cases. The demographic characteristics of patients: age and gender were collected. According to the final evaluation report of the surgeon, the clinical features related to the tumor were collected: the location of gastric adenocarcinoma; T stage (it reflects the depth of tumor invasion to gastric wall); N stage (it reflects the status of gastric lymph node metastasis); M stage (including peritoneal metastasis and other distant metastasis). Peritoneal metastasis was based on a complete surgical report of peritoneal cavity exploration. Cytological examination of peritoneal lavage fluid was performed in all cases.

### CT Acquisition Technique

The scans comprised of unenhanced chest scans and multiphase contrast-enhanced abdominal scans, which were performed to capture the cardiophrenic angle and other selected metastatic areas. A CT scanner (SOMATOM Definition Flash; Siemens Healthcare, Germany) was utilized, employing 120 kVp and 210 mAs. The entire abdomen was scanned from the thoracic entrance to the level of the inferior edge of the pubic symphysis. Nonionic contrast medium was administered with parameters of 300 mgI/ml and a flow rate of 3.0 ml/s, with a dose of 2 mL/kg based on body weight. The arterial phase and venous phase were scanned at 25 seconds and 70 seconds after contrast injection, respectively. The reconstruction thickness was set at 1.0 mm. Scans included unenhanced chest scans and multiphase contrast-enhanced abdominal scans to cover the cardiophrenic angle and other selected metastatic areas. The CT scanner (SOMATOM Definition Flash; Siemens Healthcare, Germany) was used with 120 kVp and 210 mAs, and the whole abdomen was scanned from the thoracic entrance to the level of the inferior edge of the pubic symphysis. Nonionic contrast medium parameters: 300 mgI/ml; flow rate of 3.0 ml/s; 2 mL/kg body weight. The arterial phase and venous phase were scanned at 25 s and 70 s after injection. The reconstruction thickness was 1.0 mm.

### Image analysis

Without knowledge of the surgical exploration results, two radiologists, with 7 and 21 years of experience respectively, conducted a retrospective analysis of the images in consensus. They recorded the following CT features of the cardiophrenic lymph nodes: (a) presence or absence of CPLNs, (b) dimensions in the long and short axes (if multiple lymph nodes were present, the largest one was measured), (c) side of occurrence, and (d) quantity of CPLNs. They also recorded the presence of classical signs indicating the presence of peritoneal metastasis (PM). The diameters were measured on the axial images.

Without knowing the results of surgical exploration, two radiologists in consensus (7 years and 21 years of experience) retrospectively analyzed the images. The following CT features of the cardiophrenic lymph nodes were recorded: (a) whether CPLNs appear, (b) dimensions in the long and short axes (if multiple lymph nodes are present, the largest one is measured), (c) which side and (d) the amount oCPLN. Whether there were classical signs indicating the presence of PM was also recorded. The diameters were measured on the axial images.

### Statistical Analysis

All statistical analyses were conducted using R version 4.0.5. Continuous variables were presented as medians and ranges, while categorical variables were presented as counts and percentages. Sensitivity (true positive) and specificity (true negative) were determined at the optimal cutoff probability. Receiver operating characteristic (ROC) curves were plotted. Multivariate and univariate logistic regression analyses were performed to assess the factors associated with the presence of CPLNs. A p-value of less than 0.05 was considered statistically significant. All statistical analyses were performed using R version 4.0.5.

All continuous variables were expressed as medians and ranges and categorical variables as counts and percentages. The sensitivity (true positive) and the specificity (true negative) were determined at optimal cutoff probability. We then plotted receiver operating characteristic (ROC). Multivariate and univariate logistic regression were conducted to analyze the factors associated with CPLN presence. P-values of <0.05 were considered statistically significant.

## Results

### Patient Characteristics

**Figure 1:**
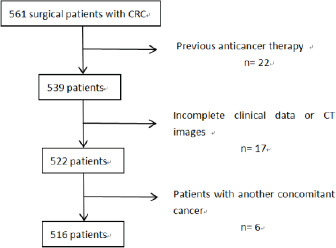
Flow chart of the study

Among the 561 patients who underwent surgery at our center between 2017 and 2019, 45 (8%) were excluded (Figure 1). The study population comprised the remaining 516 patients. Among them, 374 (72.5%) were male and 142 (27.5%) were female, with an average age of 61 years. In terms of T staging, 452 patients (87.6%) were classified as T4. A total of 203 patients (39.3%) had metastatic disease, with 134 (26.0%) exhibiting peritoneal metastasis (PM). Further details can be found in Table 1.

**Table 1:** Patient characteristics

Patients (%) N = 516	
Age (median [range]) (years)	61.0[54.0,67.0]
Sex	
Male	374(72.5)
Female	142(27.5)
Primary site	
Cardia	72(14.0)
Fundus	18(3.5)
Body	172(33.3)
Antrum	254(49.2)
T stage	
T1	10(1.9)
T2	24(4.7)
T3	30(5.8)
T4	452(87.6)
N stage	
N0	101(19.6)
N1	140(27.1)
N2	164(31.8)
N3	111(21.5)
Metastases*	203(39.3)
Peritoneum	134(26.0)
Liver	30(5.8)
Lung	1(0.2)
Retroperitoneal lymph nodes	56(10.9)
Left supraclavicular fossa lymph nodes	7(1.4)
Mediastinal lymph nodes	15(2.9)
Cardiophrenic lymph nodes	263(51.0)

Selected metatastic sites were collected, including peritoneum, liver, lung, lymph nodes (retroperitoneal, left supraclavicular fossa, mediastinum).

### CT Image Analysis

**Figure 2:**
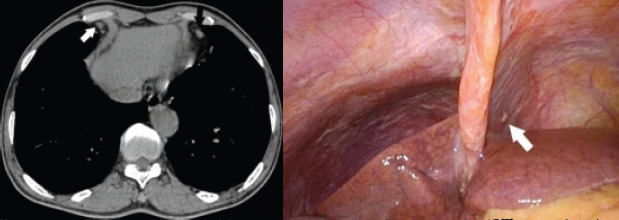
A 57-year-old male with transverse computed tomography (CT) scan showing cardiophrenic lymph nodes as the only indication of gastric peritoneum metastases

Among the 516 patients, CPLNs were identified in 263 patients (51%), with 1-5 CPLNs found per patient on average (mean: 0.8 per patient). A total of 404 CPLNs were observed, with 87% located on the right and 13% on the left side (Figure 2). The median diameter of CPLNs in the long axis was 5 mm (interquartile range [IQR]: 4-6 mm).

Out of the 134 patients with confirmed PM, 38 (28%) exhibited clear signs on CT imaging. Among the patients with clear signs, 36 (95%) had at least one CPLN, whereas 76 patients (79%) without clear signs also had CPLNs.

### Correlation between CPLN and PM

**Figure 3:**
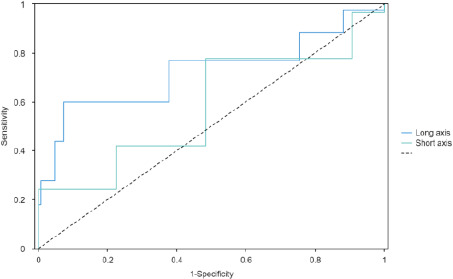
A receiver operating characteristic (ROC) curve for CPLN measuring

Among the 134 patients with PM, 112 (84%) had CPLN lesions, while among the 382 patients without PM, 151 (40%) had CPLN lesions (Table 2). For CPLNs measuring 5 mm (sensitivity [Se] 60%, specificity [Sp] 93%) (Table 3), the best accuracy was achieved through ROC curve analysis (Figure 3). Univariate analysis revealed that PM was the strongest predictor of CPLN (p < 0.001) (Table 4). A significant association was also found between CPLN and N3 lymph node status. In the multivariate analysis, PM was the only independent factor associated with CPLN (odds ratio [OR]: 7.8; 95% confidence interval [CI]: 4.6-13.0; p < 0.001). Among the 134 patients with surgically confirmed PM, 38 (28%) exhibited clear signs. CPLN occurrence was significantly higher in the 38 patients with classic signs of PM compared to the 96 patients without visible signs of PM (36/38 [95%] versus 76/96 [79%]) (Table 5). When CPLNs were considered, CT scan sensitivity increased from 28% (38/134 patients) to 85% (114/134 patients).

**Table 2:** The relationship between peritoneal metastasis status and CPLNs' amount

PM n= 134	No PM n = 382	Total n = 516	
CPLN exist(%)			
Yes	112(83.6)	151 (39.5)	263 (51.0)
No	22 (16.4)	231 (64.5)	253 (49.0)
Amount of CPLN(%)			
0	22(16.4)	231(60.5)	253 (49.0)
1	59(44.0)	119(31.2)	178(34.5)
2	23(17.2)	23(6.0)	46(8.9)
3	18(13.4)	9(2.4)	27(5.2)
4	7(5.2)	0(0.0)	7(1.4)
5	5(3.7)	0(0.0)	5(1.0)

CPLN, cardiophrenic angle lymph node. PM, Peritoneum metastases

**Table 3:** The best AUC and cut-off achieved of dimension of CPLN

AUC	Sensitivity	Specificity	Cut-off	
Long axis	0.78	0.6	0.93	5
Short axis	0.69	0.78	0.52	2

CPLN, cardiophrenic angle lymph node.

**Table 4:** Associated factors with cardiophrenic lymph nodes (CPLN)

Number of Patients	Univariate analysis		Multivariate analysis		
		OR	p	OR	p
Age					0.405
≤60	217				
>60	299	0.816	0.254	0.846	
Sex					0.422
Male	374				
Female	142	1.065	0.749	0.834	
Primary					0.998
Cardia	72				
Fundus	18	2.645	0.079		
Body	172	1.594	0.100		
Antrum	254	1.302	0.327	1.000	
T stage					0.302
T1	10				
T2	24	0.618	0.540		
T3	30	1.147	0.854		
T4	452	1.683	0.425	1.179	
N stage					0.096
N0	101				
N1	140	1.806	0.026		
N2	164	1.920	0.012		
N3	111	2.258	0.004	1.169	
Liver metastases					0.464
No	486				
Yes	30	1.276	0.521	1.349	
Lung metastases					>0.999
No	515				
Yes	1	<0.001	0.999	<0.001	
Peritoneum metastases					<0.001
No	382				
Yes	134	7.788	0.000	7.766	
OR, odds ratio					

**Table 5:** Correlation between CPLN and PM with no clear signs of PM

PM (n = 96)	No PM (n = 377)	Total (n = 473)
CPLN (%)		
Present	76	149
Absent	20	228

CPLN, cardiophrenic lymph nodes. PM, Peritoneum metastases

### Patient Characteristics

Among the 561 patients who underwent surgery at our center during 2017 and 2019, 45 (8%) were excluded (Figure 1). This study population consists of the remaining 516 patients. Among them, there were 374 (72.5%) males and 142 (27.5%) females, with an average age of 61 years. For T staging, 452 patients (87.6) were T4. There were 203 patients with metastatic disease (39.3%), of whom 134 (26.0%) had PM. Full results are reported in Table 1.

### CT-images analysis

In 263 patients (51%), 1–5 CPLNs were found (mean: 0.8 per patient). In total, 404 CPLNs were found, of which 87% were located right and 13% located left (Figure 2). In the long axis of the CPLN, the median diameter was 5 mm (interquartile range [IQR], 4–6 mm).

Among the 134 patients with confirmed PM, 38 (28%) showed clear signs. There were at least one CPLN in 36 patients (95%) with clear signs while 76 patients (79%) without clear signs.

### CPLN and PM correlation

Among the 134 patients with PM, 112 (84%) had CPLN lesions, while of the 382 patients without PM, 151 (40%) had CPLN lesions (Table 2).

For CPLN measuring 5mm (Se 60%, Sp 93%) (Table 3), the best accuracy was obtained using ROC curve analysis (Figure 3).

Univariate analysis revealed that PM was the strongest predictor of CPLN (P < 0.001) (Table 4). A significant association was also found between CPLN and the N3 lymph node status.

PM was the only independent factor associated with CPLN in the multivariate analysis (odds ratio [OR], 7.8; 95% confidence interval [CI], 4.6 ∼ 13.0; P < 0.001).

Within the 134 surgically proven PM patients, 38 (28%) had clear signs. CPLN was significantly more common in the 38 patients with classic signs of PM compared to the 96 patients without visible signs of PM (36/38 [95%] versus 76/96 [79%]) (Table 5). CT scan sensitivity rose from 28% (38/134 patients) to 85% (114/134 patients) with CPLN detection if CPLNs were considered.

## Discussion

CPLN (cardiophrenic lymph nodes) may serve as a meaningful and easily identifiable CT sign for diagnosing peritoneal metastasis in gastric cancer. Previous studies have assessed CPLN status in patients with ovarian and colorectal cancer, but there is a lack of research on CPLN presence in gastric cancer peritoneal metastases. Our study population reflects the typical incidence of gastric cancer peritoneal metastases, and as a specialized center for complex gastric cancer cases, we had a relatively higher proportion of advanced gastric cancer patients.

Our study demonstrated that CPLN was present in over half of the patients, and some patients exhibited multiple lymph nodes. Furthermore, right-sided CPLNs were more prevalent than left-sided ones. In patients without direct classical CT scan signs indicative of peritoneal metastasis in gastric cancer, the presence of CPLN was the only imaging feature in some cases. Patients with peritoneal metastases had a higher occurrence of cardiophrenic lymph nodes compared to those without the disease. The area under the curve (AUC) value corresponding to the long diameter of the cardiophrenic lymph nodes was 0.784, indicating a relatively high diagnostic value for gastric cancer peritoneal metastasis. Studies have shown that in ovarian cancer patients, the presence of enlarged lymph nodes in the cardiophrenic angle on preoperative CT scans is associated with concurrent peritoneal disease^13^,^14^. However, the criteria for lymph node enlargement lack consistency. In our study, the ROC curve revealed that a cutoff value of 5 mm provided the best judgment for the presence of peritoneal metastasis, offering a basis for defining “abnormal lymph nodes” in the cardiophrenic angle for gastric cancer patients with peritoneal metastasis. In a multivariate analysis considering demographic data and TNM staging (including factors of multiple distant metastasis), the presence of CPLN was the only factor associated with peritoneal metastasis in gastric cancer. Furthermore, combining the presence of CPLN with the typical signs of gastric cancer peritoneal metastasis obtained through CT scans significantly improved detection sensitivity, providing a non-invasive evaluation method for preoperative staging of patients.

Laparoscopy itself provides strong evidence for peritoneal metastases. The pathological basis of our study was supported by histopathological examination and exfoliated cytology of patients with peritoneal metastases detected during laparoscopy. However, as CPLN sampling was not possible during abdominal surgery, histological examination of CPLN itself could not be performed, and no studies have explored cardiophrenic lymph node sampling combined with surgery in gastric cancer patients. From an imaging perspective, cardiophrenic angle lymph nodes were easily identifiable as contrast agents were not required, and the pericardial fat provided clear contrast. Reconstruction thickness should be less than 1 mm since CPLNs themselves are small, making thin-layer reconstruction highly significant for observation and measurement. CPLN is not routinely present but can be found in patients with tumors or inflammatory diseases, serving as an important relay station for lymphatic drainage^15^,^16^. This also forms the pathophysiological basis of our study. Hypoxia-inducible factor 1 alpha (HIF-1α) has been identified as a potential diagnostic biomarker for gastric cancer peritoneal metastasis^17^. Considering its association with tumor lymph node metastasis, exploring the combined characteristics of cardiophrenic angle lymph nodes and predicting peritoneal metastasis status could be a potential direction for future research.

This study has certain limitations that need to be acknowledged. Firstly, it was a retrospective study conducted at a single center, which may introduce selection bias and limit the generalizability of the findings. The patient population at our center may not represent the overall population and could be skewed towards patients at more advanced stages of gastric cancer. Secondly, the confirmation of CPLN metastasis could not be directly established through pathological examination. Although laparoscopy provided evidence for peritoneal metastases, histological examination specifically focused on CPLN was not feasible. This aspect could be further explored in future studies by incorporating CPLN sampling during surgical procedures in gastric cancer patients. Lastly, the study did not address the prognostic significance of CPLN. Further research is needed to investigate the relationship between the presence of CPLN and patient outcomes, such as overall survival and disease progression.

## Conclusions

The detection of CPLN on CT imaging demonstrated its value as a useful tool for diagnosing peritoneal metastases in gastric cancer. However, considering the limitations of the study, further research with larger and more diverse patient cohorts is warranted to validate these findings and assess the prognostic implications of CPLN in gastric cancer. CPLN might be a meaningful and easy-to-operate CT sign for diagnosing peritoneal metastasis of gastric cancer.

Previous studies assessed CPLN status in patients with ovarian and colorectal cancer. Currently, there was a lack of studies on the presence of CPLN for peritoneal metastases of gastric cancer. Our study population was similar to the usual incidence of gastric cancer peritoneal metastases, and as a specialized center for complex gastric cancer patients, there were relatively more advanced gastric cancer patients^1^. This study showed that the presence of CPLN could be observed in over half of patients, and some patients had multiple lymph nodes, and the right cardiophrenic lymph nodes appeared much more often than the left. For those patients without direct classical CT scan signs indicative of peritoneal metastasis of gastric cancer, the presence of CPLN was the only imaging feature in some patients. Patients with peritoneal metastases had more cardiophrenic lymph nodes than patients without the disease. The AUC value corresponding to the long diameter of the cardiophrenic lymph nodes was 0.784, which meant that the long diameter had a relatively high diagnostic value for gastric cancer peritoneal metastasis. Studies had shown that for ovarian cancer patients, if preoperative CT showed enlarged lymph nodes in the cardiophrenic angle, there was more peritoneal disease at the same time^10^,^12^,^14^.

However, the criteria for lymph node enlargement were directly defined and there was a lack of consistent criteria. In this study, the ROC curve showed that 5 mm was the best cut-off value for judging the presence of peritoneal metastasis, which provided a basis for defining the “abnormal lymph nodes” in the cardiophrenic angle in patients with peritoneal metastasis of gastric cancer. In a multivariate analysis including demographic data, TNM staging (including multiple distant metastasis factors), the presence of CPLN was the only factor associated with peritoneal metastasis of gastric cancer. In addition, combining the presence of CPLN with the typical signs of gastric cancer peritoneal metastasis obtained by CT scan could greatly improve the detection sensitivity and provide a non-invasive evaluation method for evaluating the staging of patients before surgery.

Laparoscopy itself provided a high level of evidence for peritoneal metastases^8^. The pathological basis of this study was provided by the histopathological examination and exfoliated cytology of the patients with peritoneal metastases detected by laparoscopy. However, because abdominal surgery itself could not sample CPLN, the study could not perform histological examination of CPLN itself, and there was no study on cardiophrenic angle lymph node sampling combined with surgery in gastric cancer patients.

From an imaging perspective, cardiophrenic angle lymph nodes were easily identified because no contrast agent was required and the pericardial fat provided clear contrast. The reconstruction should be less than 1 mm because the CPLN itself was small, so thin-layer reconstruction was of great significance for observation and measurement.

CPLN was not routinely present but is present in some patients with tumors or inflammatory diseases, and it was an important relay station for lymphatic drainage^15^,^16^. This was also the pathophysiological basis of this study. Hypoxia inducible factor 1 alpha (HIF-1α) was a potential diagnostic biomarker for gastric cancer peritoneal metastasis^17^. Due to its relationship with tumor lymph node metastasis, its combined cardiophrenic angle lymph node characteristics to predict peritoneal metastasis status might become a potential direction for future research.

There were still some shortcomings in this study. First, a single-center retrospective study was performed. There may be selection bias in the study population, as patients at our center tended to be present at a later stage. Second, we could not directly confirm the metastasis of CPLN by pathology. Thirdly, this study did not elucidate the prognostic significance of CPLN.

In conclusion, detecting CPLN on CT was a valuable tool for diagnosing peritoneal metastases of gastric cancer.
